# Reply to Flück et al.: Alternative androgen pathway biosynthesis drives fetal female virilization in P450 oxidoreductase deficiency

**DOI:** 10.1073/pnas.2007695117

**Published:** 2020-06-23

**Authors:** Nicole Reisch, Richard J. Auchus, Cedric H. L. Shackleton, Neil A. Hanley, Wiebke Arlt

**Affiliations:** ^a^Institute of Metabolism and Systems Research, College of Medical and Dental Sciences, University of Birmingham, Birmingham B15 2TT, United Kingdom;; ^b^Medizinische Klinik IV, Klinikum der Universität München, 80336 Munich, Germany;; ^c^Division of Metabolism, Endocrinology, and Diabetes, Department of Internal Medicine, University of Michigan, Ann Arbor, MI 48019;; ^d^University of California at San Francisco Benioff Children’s Hospital, Oakland, CA 94609;; ^e^Division of Diabetes, Endocrinology, and Gastroenterology, Faculty of Biology, Medicine, and Health, Manchester Academic Health Science Centre, University of Manchester, Manchester M13 9PT, United Kingdom;; ^f^Research and Innovation, Manchester University National Health Service Foundation Trust, Manchester M13 9WL, United Kingdom;; ^g^National Institute for Health Research Birmingham Biomedical Research Centre, University Hospitals Birmingham National Health Service Foundation Trust and University of Birmingham, Birmingham B15 2GW, United Kingdom

**Fig. 1.**
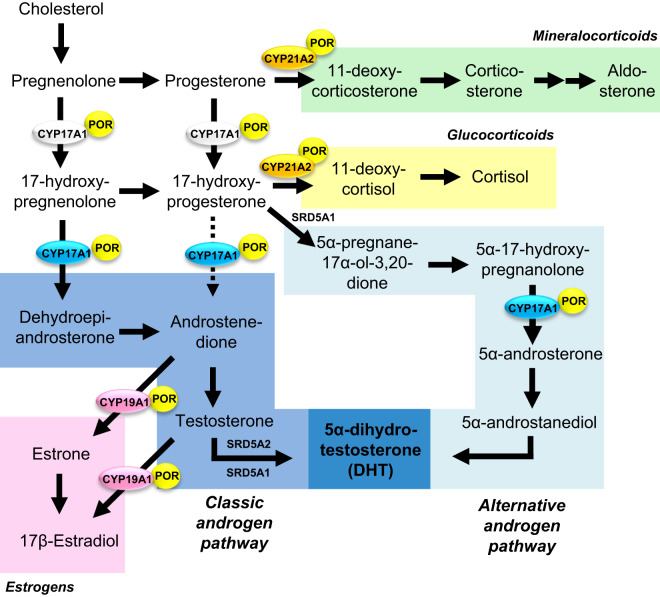
Schematic representation of steroidogenesis including the classic androgen pathway (dark blue) and the alternative androgen biosynthesis pathway (light blue), both resulting in the synthesis of potent 5α-dihydrotestosterone (DHT). Alternative pathway steroids are 5α-reduced upon pathway entry and therefore cannot be aromatized. The electron donor enzyme P450 oxidoreductase (POR) supports the activities of CYP21A2 21-hydroxylase (yellow), CYP17A1 17α-hydroxylase (white), CYP17A1 17,20-lyase (blue), and CYP19A1 aromatase (pink) in a mutation-dependent, differential manner.

Newborn girls with P450 oxidoreductase (POR) deficiency regularly present with virilized external genitalia despite low circulating androgens (1). In PNAS, we (2) explain this conundrum by enhanced prenatal activity of an alternative androgen pathway (Fig. 1) while classic androgen biosynthesis is disrupted.

Mutations in the electron donor enzyme POR invariably disrupt CYP17A1 and CYP21A2 activities (3), but have variable effects on CYP19A1 aromatase (4). We observed wild-type–equivalent CYP19A1 activity for the POR A287P mutant, within the linear range of the enzymatic reaction (2). When assessing full enzyme kinetics, Flück and coworkers (4) observed that POR A287P mildly impairs CYP19A1 activity, although catalytic efficiency was similar to wild-type POR.

Flück et al. (5) argue that mild impairment of placental aromatase activity may explain maternal virilization in POR deficiency. Our paper in PNAS (2) is primarily about fetal female virilization in POR deficiency. However, no maternal virilization was observed in three of four pregnancies with children homozygous for POR A287P (6). Maternal urine showed high excretion of alternative androgen pathway steroids, but only modest increases in the marker steroid for aromatase deficiency, 16-hydroxyandrosterone (6). Thus, impairment of aromatase activity does not appear to play a major role in maternal virilization associated with POR deficiency, although mutation-dependent variable contribution is possible.

Of note, alternative pathway intermediates are 5α-reduced (Fig. 1), and therefore nonaromatizable. In POR deficiency, classic androgen biosynthesis is disrupted, resulting in low circulating androgens (2, 7–9). Thus, with little substrate provided via the classic pathway, impaired aromatase activity will not result in clinically relevant androgen accumulation and consequently is unlikely to contribute to prenatal fetal female virilization in POR deficiency.

Flück et al. (5) question the relevance of the alternative androgen pathway in fetal female virilization, citing a recent study reporting lack of fetal adrenal expression of SRD5A1, required for alternative pathway entry. However, that paper (10) only analyzed tissues from 11 to 21 weeks postconception, after the major period of human sexual differentiation.

In our paper (2), we show that the genital phenotype depends on the differential impact of POR mutants on CYP17A1 17,20 lyase activity within the alternative pathway: Significant residual activity due to POR A287P results in virilized female newborns (46,XX DSD) and normal male genitalia. Conversely, loss of 17,20 lyase activity due to H628P causes male undervirilization (46,XY DSD) and normal female genitalia.

Flück et al. (11) previously postulated a role for AKR1C2 and AKR1C4 in the alternative pathway and that sequence variants in those enzymes cause male undervirilization. However, we (2) did not find expression of either enzyme during the major period of human sexual differentiation. It is also conceptually unlikely that disruption of the alternative pathway in the presence of normal classic androgen biosynthesis could result in male undervirilization. If this were possible, we would expect that inactivating mutations in SRD5A2, critical for the conversion of testosterone to DHT in the classic pathway, would result in normal male genitalia as the alternative androgen pathway is intact. However, SRD5A2 deficiency is a classic cause of male undervirilization.
